# Plasma therapy leads to an increase in functional IgA and IgM concentration in the blood and saliva of a patient with X-linked agammaglobulinemia

**DOI:** 10.1186/s12967-019-1928-x

**Published:** 2019-05-23

**Authors:** Jeroen D. Langereis, Joannes F. M. Jacobs, Marien I. de Jonge, Marcel van Deuren

**Affiliations:** 1 Section Pediatric Infectious Diseases, Laboratory of Medical Immunology, Radboud Institute for Molecular Life Sciences, Radboudumc, PO box 9101, 6500HB Nijmegen, The Netherlands; 2 Radboud Center for Infectious Diseases, Radboudumc, Nijmegen, The Netherlands; 3 Laboratory of Medical Immunology, Radboud Institute for Molecular Life Sciences, Radboudumc, Nijmegen, The Netherlands; 4 Department of Internal Medicine, Radboudumc, Nijmegen, The Netherlands

**Keywords:** Agammaglobulinemia, *Haemophilus influenzae*, IgA, IgM, Agglutination, Complement

## Abstract

**Background:**

Patients with X-linked agammaglobulinemia (XLA) are protected against invasive bacterial infections due to IgG replacement therapy, but are still at higher risk for mucosal infections of the gut and respiratory tract. This might be explained by to the lack of IgA and IgM, as these antibodies are especially important for protection against invading bacterial pathogens on the mucosal surface.

**Methods:**

In an attempt to eliminate a chronic norovirus infection in a patient with X-linked agammaglobulinemia, fresh frozen plasma (FFP) was given two times a week for 3 weeks. At each visit, pre- and post-FFP infusion serum and saliva was collected to determine IgG-, IgA- and IgM-concentrations and serum half-life was calculated. Functionality of the immunoglobulins pre- and post-FFP infusion in both serum and saliva was tested by measuring complement activation, agglutination and killing of non-typeable *Haemophilus influenzae* (NTHi).

**Results:**

Administration of FFP failed to eradicate the chronic norovirus infection. Serum IgA and IgM half-life was 4.2 ± 0.3 and 3.8 ± 0.3 days, respectively. The presence of serum IgM was associated with increased complement binding and complement-mediated killing of NTHi. IgA in saliva was detectable post-FFP and was associated with increased agglutination of NTHi. IgM in saliva was not detectable.

**Conclusions:**

We conclude that FFP treatment, although ineffective in clearing a chronic norovirus infection in this single patient, might be beneficial to prevent or eliminate bacterial infections in XLA patients by increasing IgM dependent complement-mediated killing in serum and IgA dependent bacterial agglutination on the mucosal surface.

## Background

Immunoglobulins are essential for defense against invading bacterial pathogens, as is clearly illustrated by the high infectious burden in patients with agammaglobulinemia such as X-linked agammaglobulinemia (XLA) [1], which is caused by mutations in Bruton’s Tyrosine Kinase (Btk) resulting in the failure of B-lymphocyte maturation [2]. The protective effect of immunoglobulins is based on three principle actions: neutralization/agglutination, opsonization and complement activation [3]. The protective activity of IgM is mainly found in the blood, of IgG in the blood and extravascular sites and of IgA at mucosal surfaces [3].

Treatment of patients with XLA patients mainly relies on IgG replacement therapy (IgGRT), which is effective in preventing invasive bacterial infections, and increases patient survival considerably [4]. However, a large proportion of patients with agammaglobulinemia still experience recurrent upper and lower respiratory tract infections [4–6]. Pneumonia in XLA patients is mainly caused by *H. influenzae* (58%), followed by *Streptococcus pneumoniae* (17%) and *Staphylococcus aureus* (17%). Chronic sinusitis is also mainly caused by *H. influenzae* (67%), followed by *S. pneumoniae* (14%) and *S. aureus* (10%) [7]. In contrast, *H. influenzae* infections are rarely seen in invasive infections such as sepsis, which is mainly caused by *Pseudomonas aeruginosa* and *S. pneumoniae* [4].

It appears that the persistent lack of IgA and IgM strongly contributes to reduced protection against invading mucosal bacterial pathogens due to deficient IgA-dependent agglutination at the mucosal surface and impaired IgM-dependent early complement activation after invasion. This is supported by examining patient cohorts with selective IgA and IgM deficiency, who have an increased risk for recurrent respiratory tract infections as well [8, 9].

Current IgGRT preparations contain IgG but are devoid of IgA and IgM [10]. There are a few preparations that also contain IgA and IgM antibodies, but none of them are used in clinical practice for the treatment of agammaglobulinemia to date [10]. In the early 1970s, fresh frozen plasma (FFP) was given to agammaglobulinemia patients. FFP infusions are well tolerated and increased serum IgG, IgA and IgM levels, however, frequent infusions are required to maintain acceptable serum antibody concentrations [11, 12].

Norovirus infection is the most frequent enteric pathogen found in patients with inherited immune deficiencies, including 2 out of 5 patients with agammaglobulinemia [13]. Treatments including increasing IVIG trough levels, enteral administration of immunoglobulins and breast milk as source of secretory IgA had limited effects in norovirus eradication [14, 15]. In recent years, we saw 5 agammaglobulinemia patients with normal supplemented IgG concentrations, but still lacking IgA and IgM, with a chronic norovirus infection. Because IgA deficiency is rather common and not associated with norovirus infection, we hypothesized that IgM might be involved in defense against norovirus because norovirus-specific IgM is detected in healthy individuals [16]. Therefore, we decided to evaluate the effect of 2 units (250–295 mL) FFP containing IgA and IgM two times a week for 3 weeks in a 22-years old XLA patient with, besides recurrent respiratory tract infections caused by *Haemophilus influenzae*, a chronic norovirus infection. During this therapy, we had the opportunity to collect serum and saliva pre-and post-FFP infusion. Serum samples were used to calculate IgA and IgM-serum half-life as well as to measure the contribution serum IgM to complement-mediated killing of non-typeable *H. influenzae* (NTHi). Saliva samples were used to determine the contribution of saliva IgA to bacterial agglutination.

## Methods

### Ethics statement

The collection of saliva was approved by the ethics committee of the Radboudumc, Nijmegen, the Netherlands (#2018-4044). Collection of blood/serum was performed for routine clinical diagnostics. All experiments were carried out in accordance with local guidelines and regulations and complies with the Declaration of Helsinki and the Good Clinical Practice guidelines.

### Serum preparation and storage

Blood was drawn in vacutainer gel serum tubes (BD Biosciences) and serum was prepared by centrifuging blood for 10 min at 4000×*g* and serum aliquots were stored at − 20 °C. Serum was thawed directly prior to use and diluted in Hank’s balanced salt solution (HBSS), without phenol red, containing Ca^2+^ and Mg^2+^ + 0.1% gelatin (HBSS3+). For complement-inactivation, serum was heat-inactivated (HI) by incubation for 20 min at 56 °C.

### Saliva preparation and storage

Unstimulated saliva was collected by spitting for 15 min in a 50 mL Falcon tube and kept on ice. Saliva was transferred to a 1.5 mL Eppendorf tube and particles were pelleted by centrifuging 10 min at 16,100×*g*. Saliva aliquots were stored at − 80 °C.

### Bacterial growth conditions and storage

NTHi strain 3655 [17] was grown in a shaker incubator at 37 °C in brain heart infusion (BHI) broth (Becton–Dickinson) supplemented with 10 μg/mL haemin (Sigma-Aldrich) and 2 μg/mL β-nicotinamide adenine dinucleotide (Merck) (sBHI) to an optical density at 620 nm = 0.5 and aliquots with 16% glycerol were stored at − 80 °C.

### IgG, IgM and IgA measurements

Serum IgG, IgM and IgA concentrations were determined by the central diagnostic laboratory of the Radboudumc on a Cobas 6000 analyzer (Roche, Basel, Switzerland) according to manufacturer’s guidelines. Serum IgA and IgM half-life was calculated using the following formula: number of days between FFP infusion/(log_0.5_ (Ig level pre/Ig level post)). Half-life was calculated from 2nd to 3rd, 3rd to 4th, 4th to 5th and 5th to 6th FFP infusion.

Saliva IgG, IgM and IgA concentrations were determined by enzyme-linked immunosorbent assay (ELISA) following manufacturer’s protocol (Thermo Fisher). For IgG, saliva was diluted 20- and 50-fold in assay buffer A. For IgA, patient saliva was diluted 10- and 20-fold in assay buffer A and control saliva was diluted 2000- and 5000-fold in assay buffer A. For IgM, saliva was tenfold diluted in assay buffer A.

### Flow cytometry

NTHi strain 3655 stock was thawed and 25 μL bacteria were added per well of a V-bottom plate and pelleted by centrifuging with 3200×*g* for 5 min. Supernatant was removed by decanting the plate and bacteria were suspended into 50 μL PBS and pelleted by centrifuging with 3200×*g* for 5 min. Supernatant was removed by decanting the plate and bacteria were suspended into 50 µL 10% HI serum supplemented with 5% agammaglobulinemia as complement source diluted in HBSS3+ for complement binding experiments, 10% HI serum or 10% HI pooled normal human serum (NHS) diluted in HBSS3+ for antibody binding experiments or undiluted saliva and incubated 30 min at 37 °C. Bacteria were pelleted by centrifuging with 3200×*g* for 5 min and supernatant was removed by decanting. Bacteria were fixed in 100 µL 2% paraformaldehyde in PBS for 20 min in at room temperature. Bacteria were pelleted by centrifuging with 3200×*g* for 5 min and supernatant was removed by decanting. All antibody incubations were performed for 15 min at room temperature in 50 µL PBS + 2% bovine serum albumin (BSA). Surface-bound complement C3 and complement complex C5b9 were detected with 1:500-diluted FITC-labelled polyclonal goat anti-human C3 (MP biomedicals) and 1:100-diluted monoclonal mouse anti-human C5b9 (Clone aE11, Santa Cruz Biotechnology) followed by 1:200-diluted PE-labelled goat anti-mouse IgG (H + L) (Thermo Fisher), respectively. Surface bound IgG, IgM or IgA was detected with 1:500-diluted Fcγ fragment-specific PE-labelled AffiniPure goat anti-human IgG (Jackson ImmunoResearch), 1:500-diluted Fc5μ fragment-specific Alexa Fluor^®^ 647 AffiniPure goat anti-human IgM (Jackson ImmunoResearch) or 1:100-diluted α-chain-specific FITC-labelled polyclonal goat anti-human IgA (Sigma-Aldrich), respectively. Surface binding of C3, C5b9, IgG, IgM and IgA was determined by flow cytometry using a FACS LSR II instrument (BD Biosciences) and expressed in mean fluorescence intensity (MFI) in arbitrary units (AU). Data were analysed by using FlowJo version 10.4.1. The percentage agglutination was determined by flow cytometry in the saliva-incubated bacterial samples as previously described [18].

### Bacterial survival assays

NTHi strain 3655 stock was thawed, pelleted by centrifugation with 16,100×*g* for 2 min. Supernatant was removed and bacteria were suspended into 1 mL PBS and pelleted by centrifugation with 16,100×*g* for 2 min. Bacteria were suspended into PBS and diluted to an OD_620_ = 0.1 in PBS and diluted 10,000-fold in HBSS3 + to obtain a concentration of ~ 200,000 CFU/mL. Fifty µL bacteria were mixed with 25 μL 40% HI patient serum day 0 pre or day 0 post and 25 μL 20% agammaglobulinemia patient serum or 25 μL 40% HI patient serum day 0 pre or day 0 post and 25 μL 20% HI agammaglobulinemia patient serum diluted in HBSS3+ and incubated 1 h at 37 °C. Samples were diluted 10- and 100-fold with PBS and three droplets of 20 μL of the undiluted, 10 and 100-fold diluted bacteria were plated on sBHI plates and grown overnight at 37 °C and 5% CO_2_. Survival was determined by dividing the colony forming unit (CFU) counts in 10% NHS by the CFU count in HI-NHS after 1-h incubation. Survival of all NTHi strains was determined in three independent experiments and the average of the three experiments was depicted in the results.

### Statistical analysis

Statistical analyses were performed with GraphPad Prism version 5.03 for Windows (GraphPad Software, Inc.). Differences were considered significant at P < 0.05. The specific statistical tests that were used for the various experiments are specified in the manuscript text or figure legends. The data is presented as mean values ± standard error of the mean, range indicate minimum and maximum values.

## Results

### Patient characteristics

The patient, now 22 years old, was diagnosed with XLA at age of 2 years. From that age he was treated with intravenous IgGRT, currently every 2 weeks (660 mg/kg) and vitamin B12 supplements. Since childhood, he experienced frequent respiratory infections, mainly caused by *H. influenza* requiring courses of antibiotics. Bronchiectasis was diagnosed at age 11 years. Next to respiratory infections, the patient experienced gastrointestinal infections with *Giardia lamblia* and *Clostridium difficile*. At the age of 17 years old, he presented with weight loss and diarrhea and stools were found positive for norovirus. The weight loss stabilized but stool samples remained positive for norovirus.

This patient was given FFP two times a week for 3 weeks as treatment to eliminate the chronic norovirus infection. However, administration of FFP did not result in the eradication of the norovirus as determined by a positive norovirus stool samples during and 1 week after treatment.

### Half-life of unmodified serum IgA and IgM in a XLA patient

**Fig. 1:**
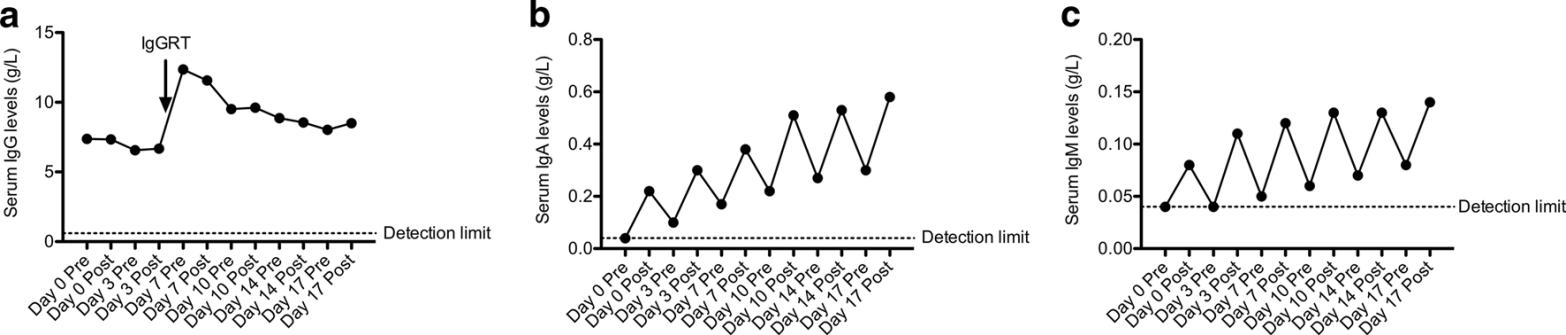
FFP treatment increases serum IgA and IgM levels. Serum IgG (**a**), IgA (**b**) and IgM (**c**) levels were determined in pre- and post-FFP treatment serum. The arrow indicates IgGRT that was given once together with the FFP treatment

Both serum IgA and IgM levels were undetectable prior to FFP infusion at day 0 (detection limit 0.04 g/L). After the first FFP infusion, serum IgA and IgM concentration increased to 0.22 and 0.08 g/L, respectively (Fig. 1b, c). At the next visit on day 3, serum IgA was decreased to 0.10 g/L, whereas serum IgM was below the detection limit of 0.04 g/L. At subsequent visits, serum IgA and IgM levels remained above the detection limit and slowly increased to 0.58 and 0.14 g/L after the last (6th) FFP infusion, respectively. Of note, these concentrations are still below the lower limit of normal serum IgA (0.7 g/L) and IgM (0.4 g/L) observed in healthy individuals [19].

Serum IgA and IgM half-life was calculated from the immunoglobulin levels post- and pre-FFP infusion as shown in Fig. 1. Half-life of IgA in this patient was 4.2 ± 0.3 days and IgM half-life was 3.8 ± 0.3 days.

### Serum IgM increases complement C3 and C5b9 binding and complement-mediated killing of non-typeable *Haemophilus influenzae*

**Fig. 2:**
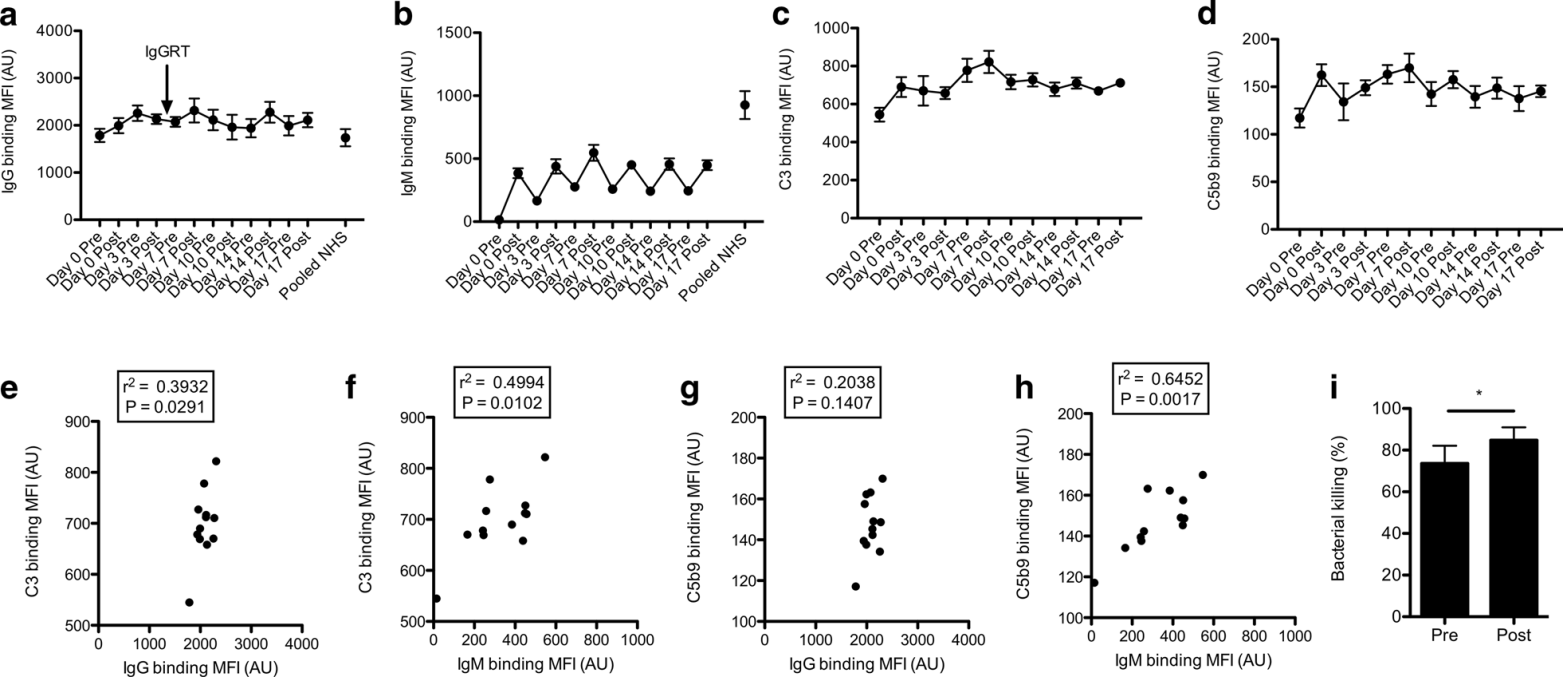
Serum IgM increases complement binding and bacterial killing. NTHi strain 3655 was incubated with 10% heat inactivated patient’s serum for 30 min and binding of IgG (**a**) and IgM (**b**) was determined by flow cytometry. Measurement of geometric mean fluorescence intensity (MFI) was depicted in arbitrary units (AU) (n = 4, mean ± standard error of the mean). The arrow indicates IgGRT that was given together with the FFP treatment. NTHi strain 3655 was incubated with 10% HI patient’s serum and 5% agammaglobulinemia patient’s serum as complement source for 30 min and binding of complement C3 (**c**) and complement complex C5b9 (**d**) were determined by flow cytometry. Measurement of geometric mean fluorescence intensity (MFI) was depicted in arbitrary units (AU) (n = 4, mean ± standard error of the mean). Correlation between binding of C3 and IgG (**e**) or IgM (**f**) was determined by linear regression. Correlation between binding of C5b9 and IgG (**g**) or IgM (**h**) was determined by linear regression. Bacterial survival in 10% HI day 0 pre- and post-FFP serum supplemented with 5% agammaglobulinemia patient’s serum as complement source was determined (n = 5). Bacterial survival is depicted in percentage compared to 5% HI agammaglobulinemia patient’s serum as complement source (n = 5, mean ± standard error of the mean). A two-tailed paired t test was used for statistical analysis. *P < 0.05 (**i**)

Binding of IgG in patient’s serum to the bacteria was relatively stable and similar to that of pooled NHS (Fig. 2a). As expected, there was no binding of IgM to the bacteria when serum was used that was collected at baseline (prior to 1st FFP infusion). Samples taken after FFP infusions showed increased IgM binding to NTHi, although less than observed when pooled NHS was used, which can be explained by the lower IgM concentration, 0.14 g/L in patient’s serum compared to 0.4–2.3 g/L as reference for normal serum IgM concentration [19].

Because both serum IgG and IgM can activate the classical pathway of complement activation, we determined complement C3 and complement complex C5b9 binding to the bacterial surface after incubation with patient’s serum. C3 and C5b9 binding increased after the 1st FFP infusions and then seemed to reach a plateau (Fig. 2c, d). Binding of C3 to the bacterial surface correlated significantly with both IgG (r^2^ = 0.3932, P = 0.0291) and IgM binding (r^2^ = 0.4994, P = 0.0102) (Fig. 2e, f), whereas C5b9 binding significantly correlated with binding of IgM (r^2^ = 0.3932, P = 0.0291) (Fig. 2g, h). Therefore, the increase in serum IgM after FFP infusion appeared to increase complement binding to the bacterial surface. In order to relate this to a functional effect, we determined bacterial killing with day 0 pre-and post-FFP infusion serum and observed significantly more killing in post- (85 ± 6%) compared to pre-FFP infusion serum (74 ± 8%).

### IgA in saliva increases agglutination of non-typeable *Haemophilus influenzae*

**Fig. 3:**
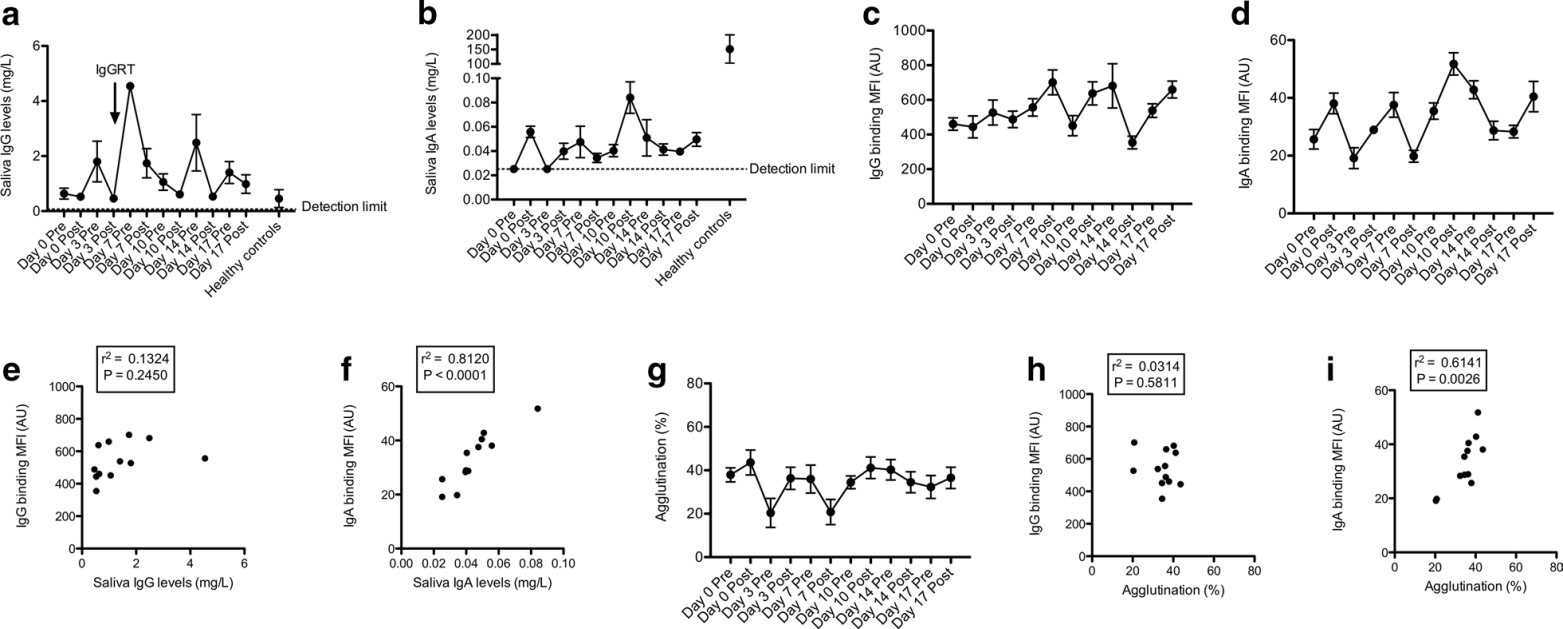
Saliva IgA increases bacterial agglutination. Saliva IgG (**a**) and IgA (**b**) was determined by ELISA (n = 4, mean ± standard error of the mean). The arrow indicates IgGRT that was given together with the FFP treatment. NTHi strain 3655 was incubated with patient saliva for 30 min and binding of IgG (**c**) and IgA (**d**) were determined by flow cytometry. Measurement of geometric mean fluorescence intensity (MFI) was depicted in arbitrary units (AU) (n = 5, mean ± standard error of the mean). Correlation between binding of saliva IgG (**e**) or IgA (**f**) to the bacterial surface and saliva IgG or IgA levels was determined by linear regression, respectively. Bacterial agglutination (**g**) was measured by flow cytometry (n = 5, mean ± standard error of the mean). Correlation between binding of saliva IgG (**h**) and IgA (**i**) to the bacterial surface and percentage agglutination was determined by linear regression

Saliva IgG in patient’s samples was variable from 0.3 to 4.5 mg/L. In three healthy controls this was 0.46 ± 0.32 mg/L (range 0.13–1.1) (Fig. 3a). Post FFP patient’s saliva IgA ranged from 0.02 to 0.08 mg/L, far lower than 152 ± 49 mg/L (range 53–206) in healthy controls and reference values (120–200 mg/L) [19] (Fig. 3b). Saliva IgM was not measurable in patient’s samples. In three healthy controls this was 3.91 ± 0.83 mg/L (2.39–5.25).

Saliva IgG and IgA bound to the bacterial surface was detectable (Fig. 3c, d). IgG levels in saliva did not correlate with binding of IgG to the bacterial surface (r^2^ = 0.1324, P = 0.2450) (Fig. 3e). However, saliva IgA levels did significantly correlate with binding of IgA to the bacterial surface (r^2^ = 0.8120, P < 0.0001) (Fig. 3f).

One of the functions of IgA is to agglutinate bacterial pathogens present on the mucosal surface [20]. Therefore, we determined bacterial agglutination with patient saliva by a flow cytometric-based assay developed in our lab [18]. Saliva samples collected before FFP infusion showed agglutination of 38% of the bacteria (Fig. 3g). Subsequent saliva samples showed differences in agglutination ranging from 20 to 44%. Saliva IgG binding to the bacterial surface did not correlate with bacterial agglutination (r^2^ = 0.0314, P = 0.5811) (Fig. 3h), but binding of saliva IgA to the bacterial surface correlated significantly with bacterial agglutination (r^2^ = 0.6141, P = 0.0026) (Fig. 3i).

## Discussion

Half-life of serum immunoglobulins have been determined frequently in the 1960s by using radio-active labelled purified serum IgM [21–24]. At first, half-life of ~ 3 days was found [22–24], but later studies using improved isolation techniques not affecting IgM functionality found a serum IgM half-life of ~ 5 days (range 3.8–6.5 days) for healthy individuals [21]. Serum IgM half-life was also determined for patients with different diseases, including agammaglobulinemia and protein losing enteropathy [21]. For 4 patients with agammaglobulinemia, serum IgM half-life was 4.2 days (range 2.2–6.5) [21], which is close to the 3.8 days found in this current study with a patient with XLA. IgM half-life was even lower for 4 patients with protein losing enteropathy, i.e. 1.4 days (range 1.0–1.7) [21]. Although not confirmed, our patient might have some degree of protein losing enteropathy because he required a high IgGRT dose (330 mg/kg/week), which might explain the lower half-life of IgA and IgM, as compared to the previous study in agammaglobulinemia [16].

FFP treatment increased serum IgA to 0.58 g/L and IgM to 0.14 g/L. As previously reported, FFP treatment used to eradicate chronic *Campylobacter jejuni* infections, increased serum IgA to detectable levels in two patients (0.42–0.56 g/L), which is in accordance to serum levels measured in our patient. In these two patients, IgM was only detectable in a single patient (0.48 g/L) [25]. In an earlier study, we showed that in vitro IgM supplementation increased complement binding and complement-mediated killing of *Campylobacter jejuni* with serum from an agammaglobulinemia patient who suffered from a C*. jejuni* spondylodiscitis [26]. In vitro supplementation of serum from an agammaglobulinemia patient with IgM also increased complement-mediated killing of NTHi [27]. In the present study, we show that supplementation of IgM in vivo by FFP treatment increases complement binding and complement-mediated killing of NTHi, which supports a role for serum IgM in complement-mediated killing of bacterial pathogens even in the presence of normal levels of serum IgG.

A proportion of XLA patients experience recurrent upper and lower respiratory tract infections, from which a large part are caused by *H. influenzae* [4–7]. This is likely due to the absence of IgA and IgM at the mucosal surface. Serum IgA and IgM can be transported to the mucosal surface through the polymeric Ig receptor [28], therefore, we assessed the saliva concentrations of these immunoglobulin classes in this patient.

Although IgG serum concentrations were hardly influenced by FFP infusion, saliva IgG differed largely, underscoring the difficulties in acquiring reproducible saliva samples. We collected saliva in a tube using an unstimulated saliva collection method for 15 min. The amount of saliva varied between 0.5 and 3 mL, which might have contributed to differences in composition between samples, and thus IgG concentrations. Although patient saliva IgG concentrations varied between samples, levels were within or above the range of 3 healthy controls. Binding of saliva IgG to the surface of NTHi was constant and did not correlate with total saliva IgG concentrations, suggesting maximal IgG binding is already achieved at all saliva IgG concentrations.

Saliva IgA was undetectable at the time of the first two FFP infusions, but became detectable at all other time points, albeit with very low concentration. Patient saliva IgA levels were almost 2000-fold lower than normal saliva IgA levels, whereas patient’s serum IgA was only fourfold lower compared to normal serum IgA levels, suggesting that most saliva IgA in healthy individuals is not transported from blood but produced locally. Our data is consistent with a study conducted more than 50 years ago [29]. In this study, 5 agammaglobulinemia patients received between 1000 and 2000 mL plasma over a period of 1 to 3 days, which led to an increase in serum IgA to 1500 mg/L and saliva IgA levels to 1 mg/L in one of these patient, much lower compared to saliva of healthy individuals (120–200 mg/L) [19].

Although saliva IgA levels were very low, we showed a strong correlation between saliva IgA binding to the bacterial surface and bacterial agglutination. Bacterial agglutination by IgA was first acknowledged by using gastrointestinal secretions and colostrum [30]. Recently, secretory IgA and secretory IgM were found to bind and agglutinate *Salmonella enterica* serovar Typhimurium, thereby limiting infection and systemic dissemination in mice [31]. Whether the IgA levels in saliva, or other mucosal surfaces such as the gut, after FFP treatment, are sufficient to prevent or treat bacterial infections is not known.

The levels of IgA and IgM were at least not sufficient to resolve the chronic norovirus infection in the gastrointestinal tract of this patient. Kempf and coworkers have tried various treatments to eradicate a chronic norovirus infection in a XLA patient, including nasogastrically administered pentaglobin containing IgM, however, without clinical response and persistent detection of norovirus by PCR [14]. Although it is thought that antibodies play a role in the clearance of norovirus infections [32], treatment with increasing IVIG trough levels, enteral administration of immunoglobulins and breast milk as source of secretory IgA had limited effects [14, 15]. We have not determined the presence of norovirus-specific antibodies in FFP, therefore, the failure for immunoglobulin preparations such as FFP in eradicating chronic norovirus infections might be due to the absence of norovirus-specific antibodies, which should be determined in future studies.

An disadvantage of FFP infusions is the frequency of administration, at least twice a week, therefore, using FFP infusions is not suitable as maintenance therapy because of the high burden for the patient. However, the use of FFP, in combination with antibiotics, might contribute in clearing chronic or severe respiratory infections when administered for a limited timeframe.

A clear limitation of this study is the use of a single patient with XLA. Therefore, we cannot firmly conclude that FFP infusion is not effective in treating chronic norovirus infection in patients with XLA. However, by collecting serum and saliva at multiple timepoints from this single patient, we were able to follow the effects of FFP infusion on IgA and IgM serum and saliva levels, as well as the functional effect of these IgA and IgM level changes. FFP infusions in this currently used treatment regimen did result in detectable serum IgM concentrations, but unfortunately not in saliva. This lack of IgM might explain the lack of effect on the norovirus infection. Clearly, more research using more patients is needed to identify strategies for the treatment of chronic norovirus infection in agammaglobulinemia patients.

## Conclusions

In conclusion, the use of serum and saliva from an XLA patient receiving IgG supplementation every 2 weeks and FFP two times a week for 3 weeks has taught us that (1) low albeit detectable serum IgA and IgM levels can be reached by frequent FFP infusions, (2) serum half-life of IgA and IgM is 4.2 and 3.8 days, respectively, (3) a small increase in serum IgM increases complement-mediated killing of NTHi, (4) FFP induces detectable concentrations of saliva IgA, which is associated with agglutination of NTHi, (5) FFP treatment in the current regimen did not result in detectable saliva IgM concentrations.

## Data Availability

Data sharing is applicable to this article.

## Abbreviations

AU: arbitrary units
BHI: brain heart infusion
Btk: Bruton’s Tyrosine Kinase
BSA: bovine serum albumin
CFU: colony forming unit
ELISA: enzyme-linked immunosorbent assay
FFP: fresh frozen plasma
HBSS: Hank’s balanced salt solution
HI: heat-inactivated
IgGRT: IgG replacement therapy
MFI: mean fluorescence intensity
NHS: normal human serum
NTHi: non-typeable *Haemophilus influenzae*
XLA: X-linked agammaglobulinemia
